# Volatile organic compounds influence the interaction of the Eurasian spruce bark beetle (*Ips typographus*) with its fungal symbionts

**DOI:** 10.1038/s41396-019-0390-3

**Published:** 2019-03-14

**Authors:** Dineshkumar Kandasamy, Jonathan Gershenzon, Martin N. Andersson, Almuth Hammerbacher

**Affiliations:** 1 Department of Biochemistry, Max Planck Institute for Chemical Ecology, Jena, Germany; 2 Department of Biology, Lund University, Lund, Sweden; 3 Department of Zoology and Entomology, Forestry and Agricultural Biotechnology Institute, University of Pretoria, Pretoria, South Africa

**Keywords:** Forest ecology, Metabolomics

## Abstract

Insects have mutualistic symbioses with a variety of microorganisms. However, the chemical signals that maintain these insect−microbe relationships are poorly known compared to those from insect−plant symbioses. The spruce bark beetle, *Ips typographus*, the most destructive forest pest in Europe, has a symbiotic relationship with several fungi that are believed to contribute to its successful invasion of Norway spruce. Here we tested the hypothesis that volatile organic compounds (VOCs) emitted from fungal symbionts could be cues for bark beetles to recognize and distinguish among members of its microbial community. Behavioral experiments with fungi showed that immature adults of *I. typographus* are attracted to food sources colonized by their fungal symbionts but not to saprophytic fungi and that this attraction is mediated by volatile cues. GC-MS measurements revealed that the symbionts emitted VOCs. Testing the activity of these compounds on beetle antennae using single sensillum recordings showed that beetles detect many fungal volatiles and possess olfactory sensory neurons specialized for these compounds. Finally, synthetic blends of fungal volatiles attracted beetles in olfactometer experiments. These findings indicate that volatile compounds produced by fungi may act as recognition signals for bark beetles to maintain specific microbial communities that might have impact on their fitness.

## Introduction

Wood-boring insects, like many other organisms, often engage in associations with symbiotic microorganisms to fulfill a range of physiological and ecological functions [1, 2]. The need of wood-boring insects for symbionts may be ascribed to the nature of their substrate, which is generally low in nutrients and high in toxic defense chemicals. In this environment, symbiont choice may be crucial for insect survival by providing essential nutrients and degrading defenses. Among wood-boring insects, many bark and ambrosia beetle species have morphological adaptations on their body, called mycangia, to carry fungi with them to new host trees and to pass on to offspring [3]. However, certain bark beetles, such as *Ips typographus*, lack mycangia. With or without mycangia, it is not clear what factors maintain the interaction between wood-boring insects and their associated fungi. One possibility is that insects create conditions in their galleries in which microbes that are important to them survive, or microbes are particularly adapted to their host tree’s environment [4–7]. However, in this scenario, some harmful microbes, such as *Ophiostoma minus* (associated with the southern pine bark beetle, *Dendroctonus frontalis*), may also take advantage of the same conditions and reduce insect fitness [8].

Symbiosis could be maintained by chemical cues. Many cross-kingdom mutualisms are driven by chemicals emitted by one or both partners [9, 10]. The response of insects to microbial signals via specialized sensory pathways represents one mode of evolutionary coadaptation that could maintain interactions with beneficial symbionts. Positive behavior towards signals from mutualists are most likely to evolve if the signals display the partner’s quality [11], as in the *Drosophila*-yeast system. These fungivorous flies utilize the volatiles produced by yeast to locate and evaluate suitable breeding sites, which maximizes benefits to their offspring [12]. Additionally, flies recognize and discriminate among different environmental microbes based on their volatile profiles, thereby maintaining a high specificity with beneficial microbes [13].

We sought evidence for signals that may be used to maintain the symbiosis between the Eurasian spruce bark beetle, *Ips typographus*, and its associated fungi. This insect is endemic to Norway spruce (*Picea abies* (L.) H. Karst.) and causes widespread damage across Europe and Asia [14]. Although it usually attacks weak and dying trees, bark beetle population sizes may increase under drought, high temperature, or forest disturbances to levels sufficient to kill mature healthy trees by mass attacks [15]. *Ips typographus* has been reported to be frequently found together with several species of fungi of which *Endoconidiophora polonica* (Siemaszko) Z.W. de Beer, T.A. Duong & M.J. Wingf., *Grosmannia penicillata* (Grosmann) Goid., *Grosmannia europhioides* (E.F. Wright & Cain) Zipfel, Z.W. de Beer & M.J.Wingf., and *Ophiostoma bicolor* R.W. Davidson & D.E.Wells are the most common [16, 17]. Other fungi such as *O. piceae* (Münch) Syd & P. Syd. are found occasionally in association with *I. typographus*, and are considered secondary symbionts [17, 18]. The association of bark beetles with fungal ectosymbionts was already recognized in 1836 [19]. However, the roles of these microorganisms in the life history of *I. typographus* and what maintains these associations remain unknown.

Here we employed behavioral assays, which demonstrated that adult beetles could differentiate among fungi and that volatile cues were involved. We used gas chromatography coupled to mass spectrometry to analyze the volatile profiles of *I. typographus*-associated fungi, and electrophysiological recordings from single sensilla on bark beetle antennae to determine which fungal volatiles were perceived. Finally, blends of synthetic fungal volatiles were tested on beetles in an olfactometer. The results support a previously overlooked role for fungal volatiles in sustaining the interaction between bark beetles and their symbiotic fungi.

## Results

### *Ips typographus* distinguishes among fungi

**Fig. 1:**
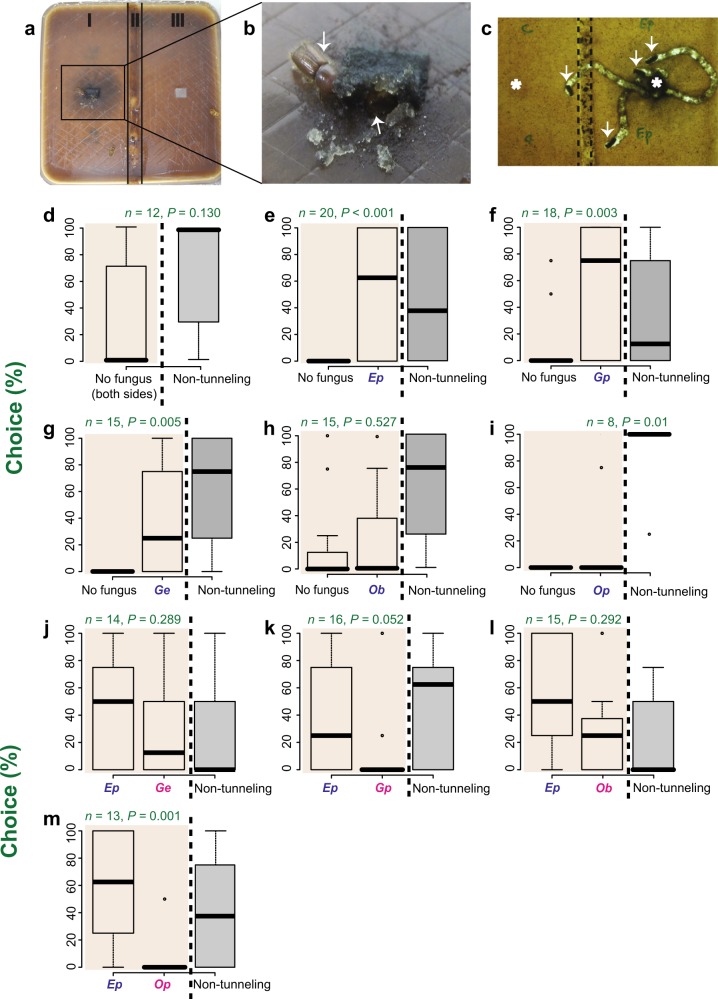
Immature adult *I. typographus* discriminate among fungal associates. **a**, **b** The bioassay arrangement consisted of a square Petri dish filled with spruce bark agar, which was divided into a fungus-colonized side (I) and a fungus-free side (III), separated by a 1 cm strip of agar containing hygromycin (II). For each trial, four callow adult bark beetles were placed into the dish. Trials with each fungal combination were replicated 8–20 times. Arrows indicate bark beetles that started to tunnel next to fungal agar plugs. **c** Enlarged bottom view of Petri dish 6 h after the addition of bark beetles. Beetles often started tunneling in or next to the mycelium on the plug. Beetles that failed to tunnel were usually found sedentary in the corner of the dish. The asterisks (*) indicates the location of agar plugs used to inoculate the medium with fungus and without fungus (control), and the arrows indicate tunneling beetles. **d**−**i** Behavioral response of beetles towards the fungus-free vs. the fungus-colonized sides. **d** There was no significant difference between tunneling and nontunneling beetles when control agar plugs (without fungus) were placed in the two sides. **e** The tunneling preference was significant when *E. polonica* (*Ep*), **f** *G. penicillata* (*Gp*), and **g** *G. europhioides* (*Ge*) were tested against the control. There was no significant tunneling preference for **h** *O. bicolor* (*Ob*) and **i** *O. piceae* (*Op*) against the control. **j**−**m** Behavioral response of beetles given a choice of two different fungi, one on each side. Beetles showed significant preference toward *E. polonica* only when this fungus was paired with *Op*

To determine if the spruce bark beetle, *I. typographus*, can recognize individual fungi frequently associated with beetle galleries and distinguish among them, we developed a behavioral assay in a Petri dish arena where callow (immature) adult beetles could choose between media colonized by different fungi for making feeding tunnels (Fig. 1a–c). After placement in the arena in the absence of fungi, beetles did not make a choice towards a specific side of the arena, and the majority did not even make feeding tunnels (tunneling vs. nontunneling beetles*, Z* = 1.51 *P* = 0.130, Wilcoxon’s test) (Fig. 1d). However, in the presence of fungi, beetles usually started to tunnel into the fungus-colonized side, starting in or next to the plugs of fungal mycelium that were used to inoculate the medium (Fig. 1b, c). Of all fungi tested (Supplementary Table S1), *E. polonica* (*Ep*), *G. penicillata* (*Gp*), and *G. europhioides* (*Ge*) were the most attractive (Fig. 1e−g) compared to the uncolonized control side (*Ep*, *Z* = 3.61, *P* < 0.001; *Gp*, *Z* = 3.01, *P* = 0.001; *Ge*, *Z* = 2.82, *P* = 0.005, Wilcoxon’s test). The side colonized by *O. bicolor* (*Ob*) was not significantly attractive to beetles compared to the uncolonized side (Fig. 1h) (*Ob*, *Z* = 0.632, *P* = 0.527, Wilcoxon’s test). Interestingly, only in assays with *O. piceae* vs. control, did adult beetles clearly avoid tunneling close to the fungus (tunneling vs. nontunneling beetles, *Z* = 2.56, *P* = 0.01, Wilcoxon’s test) (Fig. 1i), indicating an antagonistic effect of this known saprophyte towards beetles [20].

Since the galleries of bark beetles are usually colonized by more than one fungal species, our next aim was to determine whether the preference of adult beetles for tunneling near *E. polonica* changes when another fungal species was present at the other side of the arena. We found that the presence of fungi such as *O. bicolor*, *G. penicillata*, *G. europhioides*, in the same arena with *E. polonica* tended to result in a higher, but statistically nonsignificant, tunneling preference for *E. polonica* (Fig. 1j−l) (*Ep-Ge*, *Z* = 1.05, *P* = 0.289; *Ep-Gp*, *Z* = 1.94, *P* = 0.052; *Ep-Ob*, *Z* = 1.05, *P* = 0.292, Wilcoxon’s test). The presence of *O. piceae* in the same arena as *E. polonica* revealed a significant tunneling preference for *E. polonica* (*Ep-Op, Z* = 3.31, *P* = 0.001, Wilcoxon’s test) (Fig. 1m). Taken together, these results show that *I. typographus* callow adults have a strong preference for making feeding tunnels in *E. polonica*, *G. penicillata*, and *G. europhioides-* colonized diets vs. uncolonized controls, and a higher preference for *E. polonica* in comparison to the saprophyte *O. piceae*, even in a shared headspace.

### Volatiles produced by gallery-dwelling fungi are selection cues for *Ips typographus*

**Fig. 2:**
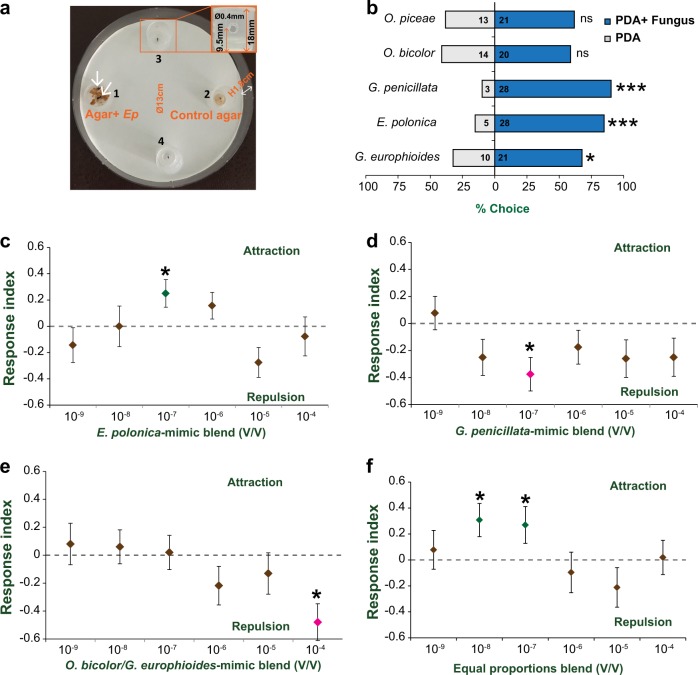
Bark beetles use volatile cues to discriminate among fungal associates. **a** The experimental setup consisted of four cups with drilled entry holes along their sides that were placed in a Petri dish (13 cm diameter). Beetles could not touch the contents of the cup prior to entering the trap, and once inside could not escape. Fungus-colonized spruce agar plugs were placed in one trap (1), spruce agar plugs without fungus were placed in the control trap (2), and the remaining two traps (3 and 4) were left empty. Arrows indicate the position of bark beetles in one trial. **b** Beetle choice between fungus-colonized agar of each species (blue bars) and control agar without fungus (white bars). The numbers inside the bars indicate the number of trapped beetles in each experiment. No beetles were trapped in the empty traps. Twenty-six trials were run with two beetles per trial. Asterisks indicate a significant difference between the fungus-colonized agar and the nonfungal control agar (Wilcoxon’s test) with ns = not significant, *P < 0.05, **P < 0.01, ***P < 0.001. **c−f** Synthetic blends of fungal volatiles found active in single sensillum recordings of bark beetle antennae were attractive or repulsive to callow adult beetles in olfactometer assays at specific doses. Blends were composed of proportional amounts of major aliphatic volatiles typical of the profile of each fungal species. Compounds included 3-methyl-1-butanol (3MB), 2-methyl-1-butanol (2MB), 3-methyl-1-butyl acetate (3MBA), 2-methyl-1-butyl acetate (2MBA), 2-phenylethanol (2PE), 2-phenylethyl acetate (2PEA), citral (CT), and citronellyl acetate (CTA). Blends were formulated as follows: **c** *E. polonica* blend (3MB:2MB:3MBA:2MBA:2PE:2PEA:CT:CTA, 10:15:200:70:1:30:2:10), **d** *G. penicillata* blend (3MB:2MB:3MBA:2MBA:2PE:2PEA, 30:15:2:1:12.5:25), **e** *O. bicolor*/*G. europhioides* blend (3MB:2MB:2PE, 2:2:1), and **f** equal proportions blend (3MB:2MB:3MBA:2MBA:2PE:2PEA, 1:1:1:1:1:1). Scale response index extends from −1 (full avoidance) through 0 (neither attraction nor avoidance) to +1 (full attraction). Response index (RI) for each dose was determined using 52 beetles with two beetles per replicate (*n* = 26), and the deviation of RI against zero was tested using Wilcoxon’s test. Asterisks denote significant differences, *P < 0.05. Error bars indicate SEM

We performed a bioassay where beetles could distinguish food sources only through the volatiles they produced (Fig. 2a). Using 4-day-old cultures, we found that callow adult beetles were attracted to volatiles originating from three species of fungi (compared to no fungus controls): *E. polonica*, *G. penicillata*, and *G. europhioides*, with stronger attraction towards *E. polonica* (*W* = 14, *P* = 0.0004, Wilcoxon’s test) and *G. penicillata* (*W* = 14.5, *P* = 0.0009, Wilcoxon’s test) (Fig. 2b) compared to *G. europhioides* (*W* = 30, *P* = 0.043, Wilcoxon’s test). On the other hand, volatile compounds originating from *O. piceae* and *O. bicolor* did not attract or inhibit the beetles (Fig. 2b, *Op*, *W* = 52.5, *P* = 0.251; *Ob*, *W* = 45, *P* = 0.396; Wilcoxon’s test). These results show that callow adult *I. typographus* may use volatile organic compounds originating from fungi to distinguish between them.

### Volatile organic compounds are emitted by fungi frequently found in association with *Ips typographus*

As we observed a strong preference of bark beetles to volatiles from certain fungi, we sampled the headspace of each fungal species and identified the major volatile compounds by gas chromatography and mass spectrometry. The volatile profile of each fungal species was unique (as represented in a principal component analysis, Supplementary Fig. S1), and varied both qualitatively and quantitatively, and with fungal development (Supplementary Table S3-S8).

**Fig. 3:**
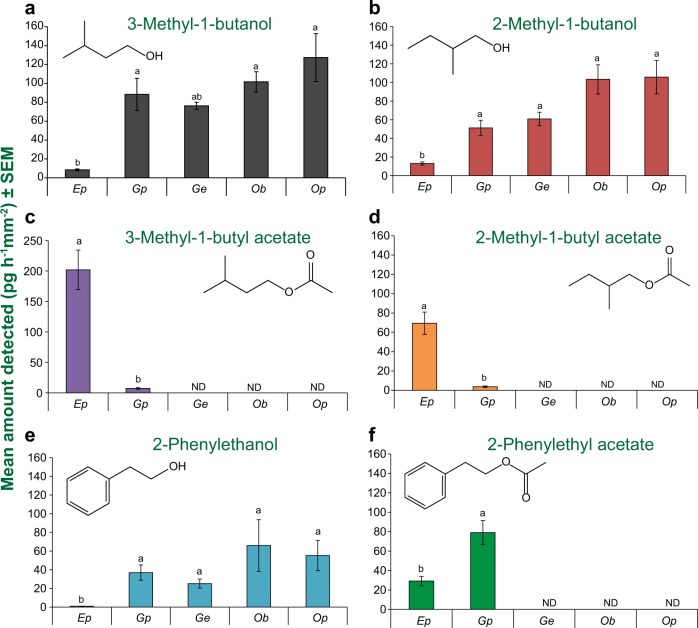
Major alcohols and esters emitted by beetle-associated fungal species. *Endoconidiophora polonica* emitted higher levels of acetate esters and lower levels of the parent alcohols than the other species. Error bars indicate SEM. Significant differences between species are denoted by different lowercase letters (*n* = 5, ANOVA, Tukey’s test, *P* < 0.05)

Among the major compounds, all species emitted the branched-chain C_5_ alcohols, 3-methyl-1-butanol and 2-methyl-1-butanol, and the aromatic alcohol 2-phenylethanol (Fig. 3), but *E. polonica* released significantly lower amounts compared with other fungi (2-PE, *F*_4,20_ = 27.29; 3-MB, *F*_4,20_ = 7.46; 2-MB, *F*_4,20_ = 18.82 ANOVA, Tukey’s test, *P* < 0.05) (Fig. 3a, b, e). Instead, *E. polonica* emitted the corresponding acetate esters (Fig. 3c, d), with 2-phenylethyl acetate also dominant in *G. penicillata* (Fig. 3f). The volatile profiles of all of the associated fungi, except *O. bicolor*, contained terpenes with major differences among the species. *Endoconidiophora polonica* emitted an assortment of acyclic monoterpenes and sesquiterpenes, such as citral, geranyl acetate, citronellyl acetate and 2,3-dihydrofarnesyl acetate (Supplementray Table S4). *Grosmannia penicillata* and *G. europhioides* released the bicyclic sesquiterpenes, (*E*)-β-caryophyllene and caryophyllene oxide, at high rates, while *O. bicolor* emitted no major terpenes (Supplementary Table S5, 6, 8). The saprophyte *O. piceae* emitted large amounts of the tricyclic 6-protoilludene and various unknown sesquiterpenes (Supplementary Table S7). These volatile profiles of the fungi investigated are similar to those of related fungi previously described in the literature [21–23]. These results demonstrate that each fungal bark beetle symbiont tested emits a distinct blend of volatiles, although a few compounds are produced in common.

### Single sensillum recordings from bark beetle antennae reveal olfactory sensory neurons for fungal compounds

**Fig. 4:**
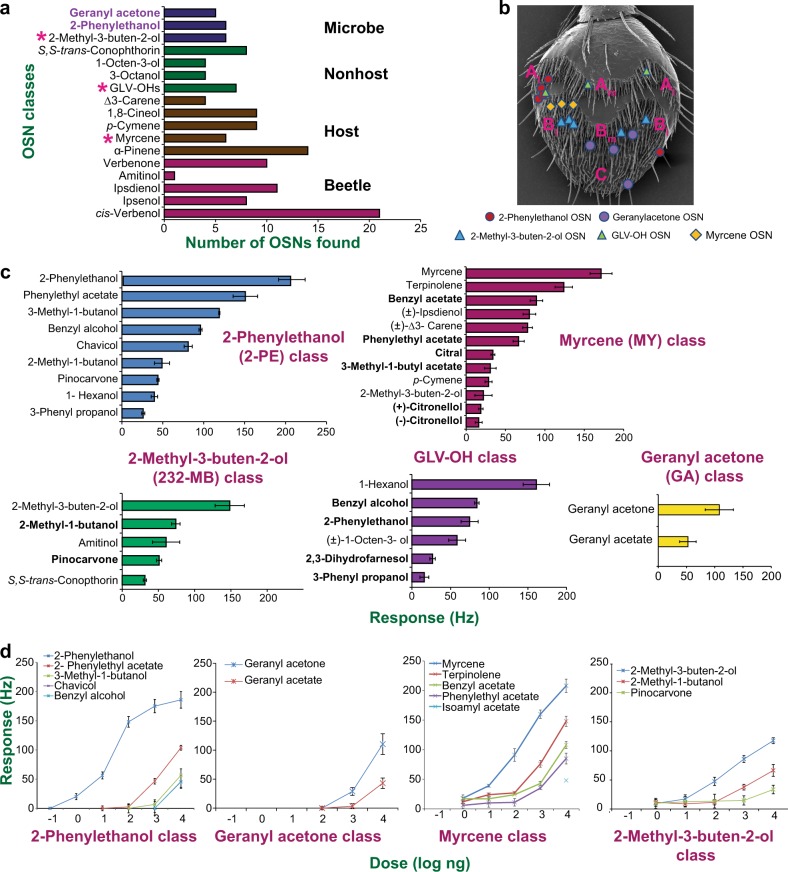
Single sensillum recordings from *I. typographus* antennae demonstrate beetle detection of fungal volatiles. **a** Number of olfactory sensory neurons (OSNs) identified of each class. OSN classes were named according to the compound that elicited the strongest response [24]. The two newly discovered classes responsive to fungal compounds are indicated in purple text. Asterisks denote previously described OSN classes [24] that were found to respond to a lesser extent also to fungal compounds. Listed on the right are the major sources of odors to which each OSN class primarily responds. **b** OSN classes responding to fungal volatiles have discrete distributions on the beetle antenna. Olfactory sensilla are distributed in three main regions denoted A, B, and C [52], of which regions A and B are divided into medial (m) and lateral (l) subregions. **c** Response spectra of OSN classes responding to fungal volatiles (10 µg dose). Fungal volatiles are listed in bold. **d** Dose−response curves of OSN classes responding to fungal volatiles: 2-PE (*n* = 8), GA (*n* = 6), MY (*n* = 2), 232 MB (*n* = 5). Error bars in (**c**) and (**d**) represent SEM. 2-PE 2-phenylethanol, GA geranyl acetone, MY myrcene, 232-MB 2-methyl-3-buten-2-ol

To identify which fungal volatiles are detected by *I. typographus*, we performed single sensillum recordings (SSR). We screened 212 randomly chosen sensilla across the antennal surface of 32 beetles (14 males and 18 females), using an odor panel comprising 85 compounds (Supplementary Table S2). Based on odor response profiles, a large number of olfactory sensory neurons (OSNs) could be matched to 15 of the previously characterized OSN classes in this species [24]. In addition, two new OSN classes were discovered, which primarily responded to the fungal volatiles 2-phenylethanol and geranyl acetone, respectively and with nonrandom distribution patterns across the antenna (Fig. 4a, b).

Six OSNs responded most strongly to 2-phenylethanol with secondary responses elicited by other fungal volatiles, including 2-phenylethyl acetate, 3-methyl-1-butanol, benzyl alcohol, 2-methyl-1-butanol and 1-hexanol (Fig. 4c). OSN responses of *I. typographus* to 2-phenylethanol had been noted previously, but the specificity and sensitivity of this OSN class had not been characterized [25]. The response to 2-phenylethanol was above 200 Hz at the 10 µg dose (Fig. 4d), and the response threshold for this compound (ca. 1 ng compound on the filter paper) was 100-fold lower than that for the second most responsive compound, 2-phenylethyl acetate.

We also identified five OSNs that primarily responded to the fungal compound geranyl acetone, followed by geranyl acetate (Fig. 4a, c). The response threshold for geranyl acetone was between the 100 ng and 1 µg doses, while geranyl acetate failed to elicit responses below 10 µg (Fig. 4d). The previously identified OSN class tuned to the aggregation pheromone component 2-methyl-3-buten-2-ol (232-MB) [24] was here also shown to respond to the structurally similar fungal volatile 2-methyl-1-butanol, and less so to a few other compounds including pinocarvone (Fig. 4c). The response threshold for 232-MB was clearly lower than that for 2-methyl-1-butanol. 232-MB has been reported from adult bark beetles and from fungi [26, 27] (Fig. 4d). Some fungal volatiles also elicited secondary responses in previously characterized OSN classes that respond primarily to nonfungal compounds. For example, the OSN class which is most sensitive to myrcene (a host tree monoterpene), responded secondarily to the fungal volatiles benzyl acetate, 2-phenylethyl acetate, isoamyl acetate and citral. The OSN class known to respond primarily to three repellant green leaf volatiles [24, 28, 29] was also somewhat excited by 2-phenylethanol, benzyl alcohol, 1-octen-3-ol and weaker responses were induced by (-)-2,3-dihydrofarnesol and 3-phenyl propanol at 10 µg doses (Fig. 4c (GLV-OH)).

Taken together, our results demonstrate that volatiles from fungal symbionts are detected by dedicated OSNs in *I. typographus* as well as OSNs primarily responding to compounds originating from other sources. Thus, the odors of fungi appear to be at least partly encoded by a combinatorial mechanism [30].

### Synthetic blends of SSR-active fungal volatiles attract or repel bark beetles in a dose-dependent manner

To determine if the compounds that elicited electrophysiological responses were also behaviorally active when tested with callow adult beetles in an olfactometer, we formulated synthetic blends mimicking each fungus. Of all fungus-mimicking blends tested, only the *E. polonica* eight-component blend at a dose of 10^−7^ was significantly attractive to the beetles compared to the heptane control (Fig. 2c) (*W* = 212.5, *P* = 0.031, Wilcoxon’s test). Neither the *G. penicillata* six-component blend, nor the *O. bicolor* three-component blend, was attractive at any dose tested (Fig. 2d, e). The 10^−7^ dose and 10^−4^ dose of the *Gp* and *Ob* blends, respectively, were even repulsive to walking beetles (*Gp* 10^−7^ dose, *W* = 112.5, *P* = 0.016; *Ob* 10^−4^ dose, *W* = 178, *P* = 0.0043, Wilcoxon’s test) (Fig. 2d, e). The beetles’ negative behavior towards the *G. penicillata* six-component blend was unexpected because the volatiles from the whole fungus were attractive (Fig. 2b). In fact, the response indices of the majority of blends and doses tested were in the negative region, although most were nonsignificant (Fig. 2d). Therefore, we formulated a synthetic blend with equal ratios of five components that are shared by both *E. polonica* and *G. penicillata* to determine if the ratios of different components in the blend influence the valence of the whole blend. This blend significantly attracted beetles at two doses (10^−7^ and 10^−8^) (Fig. 2f) (10^−7^, *W* = 54.5, *P* = 0.050; 10^−8^, *W* = 54, *P* = 0.028, Wilcoxon’s test).

## Discussion

Many aggressive beetles infesting conifers over a wide geographical range [15, 31, 32] are associated with fungal symbionts, which are phylogenetically related to each other. However, bark beetle-associated fungi occupy the same niche as wood-inhabiting saprophytes, and compete for the same resources and even for the beetle vector itself [33]. Despite this, mutualistic bark beetle symbionts are consistently transmitted between beetle generations, indicating the presence of mechanisms that maintain this association. Similar to other species of bark beetles, *I. typographus* co-occurs with several species of fungi that contribute to successful host tree invasion [10, 16, 17]. Here we investigated *I. typographus*, newly emerged from their pupae that engage in a feeding period in the host tree in which they were raised prior to their search for a new host. We show that these callow adults use olfactory cues to select among the fungal species commonly occurring in the host tree and excavate feeding tunnels nearby. Electrophysiological investigations show that this beetle can detect many of the major compounds found in the headspace of co-occurring fungi, and some of its OSNs are even specialized on fungal volatiles. Finally, callow adult beetles were attracted to specific blends and concentrations of fungal headspace volatiles. Depending on the mutualistic role of *I. typographus*-associated fungi, which is still under debate [33], newly emerged beetles may be using volatiles during this phase as cues to detect bark zones of high nutritional value or to choose fungi to disperse with that can best help overcome the defenses of its new host tree.

Fungal colonization of bark beetle galleries is highly unpredictable and depends on several factors including interspecific competition, defense status of the tree, moisture content of the substrate and local temperature [10, 18, 34]. In addition to bark beetles as vectors for ophiostomatoid fungi, phoretic mites associated with bark beetles are known to carry hyperphoretic ascospores of many ophiostomatoid fungi, especially *O. bicolor* [18, 35]. This fungus was in our assays less attractive to the beetle than *E. polonica, G. penicillata and G. europhioides*, the most frequent associates of *I. typographus*. Furthermore, the antagonistic effects of *O. piceae* on tunneling callow adults might explain the low incidence of this fungus in natural populations [16–18]. Consistent transmission of specific fungi might therefore not only rely on the competitive advantage of more virulent, well-adapted fungi [36], but might also rely on active beetle selection during its maturation phase.

Only fungi that emitted higher amounts of the volatile esters, 3-methyl-1-butyl acetate, 2-methyl-1-butyl acetate and 2-PEA were highly attractive to beetles, and the attraction varied with the composition of the blend. Several insect species are attracted to mixtures containing esters. For example, esters produced by *Saccharomyces cerevisiae* are attractive to vinegar flies which benefit nutritionally by dispersing this microorganism [37, 38]. Previous studies on bark beetles also provide ample precedent for our findings. For example, larvae of the walnut twig beetle, *Pityophthorus juglandis*, were attracted to the volatiles emitted by their symbiotic fungus, *Geosmithia morbida*, and several other nonsymbiotic fungi, but adults were not [39]. The red bay ambrosia beetle, *Xyleborous glabratus*, was also attracted to the volatiles from its fungal symbiont *Raffaelea lauricola*, which is a primary food source for the beetle, but the beetle was not attracted to volatiles from nonsymbiotic fungi [40]. Although blends of fungal volatiles have not been tested with conifer bark beetles previously, there is already evidence for the roles of individual volatiles in attraction. For example, the attraction of *Dendroctonus frontalis*, the southern pine beetle, to an unattractive pheromone blend increased when low concentrations of the fungal volatiles 2-phenylethyl acetate and 3-methyl-1-butyl acetate were added [41], but 2-PE inhibited the attraction. The attraction of the coffee bean weevil to cultures of its symbiont, *Kluyveromyces lactis*, is based on compounds such as 2-PE and 2-PEA [42]. However, volatiles released by fungi under natural conditions in the field have not been studied yet and would shed further light on our findings, especially with regards to the narrow range of concentrations that were behaviorally active in our bioassays.

The nature of the symbiosis between *I. typographus* and its associated fungi is still unclear. While the fungi benefit from being transported by the beetle to a new host tree and being inoculated in a freshly dug entry hole, the beetle may accrue nutritional benefits by feeding directly on the fungus [43]. The strong preference of newly enclosed beetles in our fungal choice assays to feed next to agar colonized by the preferred fungus supports a nutritional role. Beetles that ingest more nutrients in a shorter period can disperse more rapidly to new host trees. Newly emerged adults of *Dendroctonus ponderosae* that fed on the symbiont *Grosmannia clavigera* made shorter feeding galleries than adults feeding on fungus-free tissue because they encountered more nutrients [44]. Fungi may also benefit beetles by degrading host tree defenses that are toxic to beetles [5, 45, 46]. *Endoconidiophora polonica* and *G. penicillata* are also reported to be highly virulent spruce pathogens and thus may also benefit beetles by hastening tree death, especially early in the colonization phase [6, 46, 47].

The ability of fungal volatiles to elicit behavioral responses in bark beetles implies that they may be useful in managing outbreaks of these destructive insects [10]. Control of bark beetle populations with pheromones has long been viewed as an environmentally responsible alternative to the use of synthetic insecticides. Attractive volatiles produced by beneficial fungal symbionts might be employed in combination with pheromones in mass trapping of beetles. Alternatively, repellant volatiles from other fungal associates could augment known repellant compounds [28] to deter bark beetle attack. In either case, our developing knowledge about chemically mediated interactions between bark beetles and their associated fungi could assist in developing new approaches to control these destructive forest pests.

## Materials and methods

### Fungal strains, chemicals and culture medium

The common fungal associates of *Ips typographus* used in this study are listed in Supplementary Table S1. All isolates were maintained on 4% potato dextrose agar (PDA; Sigma Aldrich, MO, USA) at 4 °C and subcultured in fresh medium for 3–5 days before use in experiments. Spruce bark agar was prepared from 7% finely ground inner bark from noninfested logs (w/v) mixed with 4% agar and dispensed in Petri dishes after heat sterilization. All chemicals used are listed in Supplementary Table 2.

### Bark beetle rearing and storage

For the behavioral bioassays, bark beetles were reared in 30 cm diameter × 50 cm height freshly cut spruce logs enclosed in an insect tent kept in a climate chamber programmed to 25 °C, relative humidity 65% and a photoperiod of 20 h per day. After approximately 45 days, immature or so-called callow adults were carefully collected by manually removing bark from logs and kept at 4 °C in Petri dishes containing moistened towel paper for a few days. For long-term storage of beetles, fresh bark pieces were inserted into clean 15 mL Falcon tubes lined with moistened filter paper, and 50 adult beetles were stored in each tube. After 8−12 h, the tubes containing actively tunneling beetles in the fresh bark tissue were maintained at 4 °C. Only callow adult beetles were used in behavioral assays and naturally emerged mature beetles were used for rearing.

### Fungal choice assay

Square Petri dishes with 7% spruce bark agar were divided into two sides separated by a strip amended with 100 µg mL^−1^ hygromycin (Fig. 1a). The experimental setup consisted of either control vs. fungus-colonized sides of the dish or with the two sides colonized by different fungi. The control side received a 10 mm fungus-free agar plug whereas each fungal side was inoculated with a mycelium plug from a culture of an actively growing fungus. Fungi were allowed to colonize the medium for 2–3 days prior to the assay. Adult bark beetles were surface sterilized in 1.2% bleach (from 12% NaClO in H_2_O; Carl Roth, Germany) and 2% ethanol for 30 s followed by three rinses in sterile water. Four active adult beetles were placed on each plate and the plate was stored in darkness at 25 °C. Side selection and tunneling preferences of beetles were monitored for up to 12 h and denoted as percentage choice of beetles tunneling or nontunneling in each plate. Each plate contained at least one male and one female beetle. Occasional beetles tunneling in the cracks along the edges of the dish were excluded from the analysis. The first batch of tunneling preference assays were performed in August 2015 and repeated again in January 2019. Both datasets were combined as results were similar. Differences in the percentage of tunneling beetles in control vs. fungus-colonized diets, as well as tunneling vs. nontunneling beetles in each plate, were analyzed using the Wilcoxon nonparametric test using the SPSS software (version 17). In assays with *O. piceae* vs. control, beetles did not tunnel next to the *O. piceae* colonized diet (Fig. 1i). Hence, the difference in the tunneling behavior between the control side and the *O. piceae* side could not be analyzed statistically.

### Fungal volatile choice assay

The experimental setup consisted of a 13 cm diameter circular Petri dish with four circular plastic cups attached to the base equidistant from each other and 2 cm from the outer edge of the bottom dish (Fig. 2a). The plastic cups (1.8 × 1.8 cm, diameter × height) were mounted on thumbtacks with the open end of the cup facing the lid of the Petri dish. Four 0.4 mm Ø holes at a distance of 0.9 cm from the bottom were drilled in the sides of each cup such that the beetles could enter the cup but not leave. The lid of the Petri dish was secured tightly so that beetles could enter the cups only through the side holes and not from the open end of the cups. Additionally, eight small pinholes were made on the walls of the Petri dish to facilitate air diffusion. The inner surface of the Petri dish was covered with a filter paper to prevent beetles from slipping. Plugs (10 mm in diameter) of 2% PDA with a 4-day-old culture of actively growing fungal mycelium (or without mycelium as controls) were placed inside the circular cups opposite each other. The other two cups were left empty as controls. Two beetles were placed on each plate and the plate kept in a laminar flow hood in darkness at 25 °C. Choices were monitored periodically up to 6 h. Chemotaxis indices (CI) were calculated using the formula: ${\mathrm {CI}} = {\textstyle{{{\mathrm {number}}\,{\mathrm {of}}\,{\mathrm {beetles}}\,{\mathrm {in}}\,{\mathrm {treatment}}\,{\mathrm {trap}}\,-\,{\mathrm {number}}\,{\mathrm {of}}\,{\mathrm {beetles}}\,{\mathrm {in}}\,{\mathrm {control}}\,{\mathrm {trap}}} \over {{\mathrm {total}}\,{\mathrm {number}}\,{\mathrm {of}}\,{\mathrm {beetles}}}}}$. The CI values from each treatment were analyzed by Wilcoxon’s signed rank test to compare the differences between treatments within groups using R package (version 3.3.1).

### Volatile collection from fungi

Actively growing fungi (Supplementary Table S1) were inoculated on PDA, and headspace volatiles were collected after 4 days of incubation in darkness at 25 °C. A single Petri dish (control or fungus inoculated) was placed inside a 1 L volatile collection glass jar with the Petri dish lid open slightly (~5 mm) to facilitate diffusion of volatiles. Active charcoal-filtered air was pumped into the collection jar and the outgoing air was passed through a filter packed with 150 mg Super Q adsorbent at a flow rate of 0.45 L min^−1^ for 24 h. After each experiment, the surface margin of fungus on the PDA plate was photographed and measured using ImageJ software. Volatile traps were eluted twice with 200 µL dichloromethane spiked with 10 ng µL^−1^ nonyl acetate (Sigma Aldrich) as an internal standard.

### Quantitative volatile analysis of fungal volatiles by gas chromatography-mass spectrometry and flame-ionization detection

The eluent from the traps was subjected to gas chromatography using an Agilent 6890 series gas chromatograph (Agilent, Santa Clara, CA, USA) with injection 1 µL splitless and flow, 2 mL min^−1^. The constituents were separated on a DB-5MS column (Agilent, 30 m × 0.25 mm × 0.25 µm) with a temperature gradient of 45 °C to 180 °C at 6 °C min^−1^ and then a rise to 300 °C at 100 °C min^−1^. For compound identification, the column outlet flow (He as carrier gas) was coupled to an Agilent 5973 quadrupole mass selective detector with interface temperature 270 °C, quadrupole temperature 150 °C, source temperature 230 °C and electron energy 70 eV. The identity of each peak was determined by comparison of its mass spectrum and retention time to those of authentic standards or spectra in reference libraries (NIST98 and Wiley 275). For compound quantification, the column outflow (H_2_ as carrier gas) was coupled to a flame-ionization detector set at 300 °C. The amount of each compound was calculated from the peak area obtained in comparison with that of the internal standard and standardized to the culture surface area (due to differences in fungal growth rates [36]) with normalized response factors for each compound. Normality of data was assessed using the Shapiro−Wilk test and non-normally distributed data were transformed using the natural logarithm. Differences between the emissions of volatiles from different fungal treatments were tested using ANOVA followed by Tukey post-hoc test using the laercio package in R.

### Semi-quantitative analysis of volatiles over fungal culture development

Volatiles were collected at different time intervals after culture inoculation (1, 4, 8, and 12 days) using polydimethylsiloxane (PDMS) sorbent [48]. Two to three 5 mm PDMS tubes were carefully mounted on sterile metal wires imbedded in the PDA. The headspace sampling time was 2 h for all experiments unless otherwise indicated. After sampling, the tubes were placed in 1.5 mL brown glass vials and stored at −20 °C for analysis.

Volatiles collected on PDMS tubes were analyzed using a GC-2010 plus gas chromatograph coupled to a MS-QP2010 quadrupole mass spectrometer equipped with a TD-20 thermal desorption unit (Shimadzu, Japan) and a GC Cryo-Trap (Tenax®). A single tube was placed in a 89 mm glass thermal desorption tube and desorbed at a flow rate of 60 mL min^−1^ for 8 min at 200 °C under a stream of N_2_ gas. The desorbed substances were focused in a cryogenic trap at −60 °C. The Tenax® adsorbent was heated to 230 °C and the analytes were injected using split mode (1:50) onto a Rtx-5MS GC column (thickness—0.25 µm, length—30 m, film diameter—0.25 µm) using helium as carrier gas. Separation, detection, and data analysis were identical to those reported for the quantitative analysis.

### Single sensillum recordings

Adult bark beetles used in electrophysiological experiments originated from a laboratory colony that was reared under the conditions described in ref. [49], and kindly provided by Prof. F. Schlyter (SLU, Alnarp, Sweden). An antenna attached to a living *I. typographus* was mounted in dental wax on a coverslip placed on a microscope slide [24]. Then, SSR using electrolytically (KNO_2_) sharpened tungsten microelectrodes and standard equipment (Syntech, Kirchzarten, Germany) was conducted as previously described [50]. Measurements were performed on 212 randomly chosen sensilla across the antennal surface of 32 beetles (14 males and 18 females). The sex of each beetle was determined by dissection of genitalia after the recordings.

The odor panel comprised 85 compounds, including several newly identified fungal volatiles (Supplementary Table S2). Pheromone, host, and nonhost compounds that were previously reported as electrophysiologically active on *I. typographus* were included as diagnostic odorants for OSN classes already described ([24]; Supplementary Table S2). All compounds were diluted in paraffin oil (w/v) except for *cis*-verbenol and 2,3-dihydrofarnesol, which were first diluted in tetrahydrofuran (THF) and hexane, respectively, with relevant control stimuli also tested. Odor stimuli (10 µL) were applied on filter paper strips (1.5 × 0.5 cm) placed inside Pasteur pipettes and capped with 1 mL plastic pipette tips. Odor pipettes were used for a maximum of eight consecutive stimulations for screening and two stimulations for dose−response assays, and stored at −18 °C between recordings [51]. To facilitate comparisons with previous SSR studies of *I. typographus*, we did not correct stimulus loads with respect to differences in compound volatility. During screening experiments, the compounds were tested in random order (dose: 10 µg on the filter paper). In subsequent dose−response assays performed on additional OSNs, compounds were tested at increasing doses from 10 pg to 10 µg on the filter paper. To avoid sensory adaptation, weakly active compounds were tested before more potent ones, and OSN firing was always allowed to return to baseline (spontaneous) activity between consecutive stimulations.

Autospike 3.0 (Syntech) was used to count the number of spikes for the first 0.5 s of the response. The number of spontaneous spikes during the immediate 0.5 s pre-stimulation period was subtracted from the number of response spikes, and the resulting net response converted to spikes s^−1^ (Hz). In a few cases when the blank control elicited spike activity, the response to blank was subtracted from the odor response. During screening at the high (10 µg) dose, any net response below 20 Hz was regarded as “no-response”.

### Beetle bioassay with volatile blends

These assays used the same experimental setup as described above, except that blends of synthetic compounds diluted in heptane were used. For each blend, 10 µL was applied to 10 mm Whatmann filter paper laid on the top of PDA plugs placed inside the cups. The solvent was allowed to evaporate for 5 min before starting the assay. Fungal species-specific blends were formulated using compounds that elicited OSN responses in the beetle. The ratio of each compound in the mix was calculated from the GC-FID profiles (Supplementary Table S3). Further dilutions in log_10_ steps were made by dissolving in heptane. Two active beetles were placed in each plate and the entire setup was kept in a fume hood in darkness at 25 °C with beetle choices monitored periodically up to 6 h.

## Supplementary information

Supplemental figure 1

Supplemental table 1

Supplemental table 2

Supplemental table 3

Supplemental table 4

Supplemental table 5

Supplemental table 6

Supplemental table 7

Supplemental table 8

## References

[1] Hansen AK, Moran NA (2014). The impact of microbial symbionts on host plant utilization by herbivorous insects. Mol Ecol.

[2] Janson EM, Stireman JO, Singer MS, Abbot P (2008). Phytophagous insect-microbe mutualisms and adaptive evolutionary diversification. Evolution.

[3] Francke-Grosmann H (1967). Ectosymbiosis in wood-inhabiting insects. Symbiosis.

[4] Wang Y, Lim L, Madilao L, Lah L, Bohlmann J, Breuil C (2014). Gene discovery for enzymes involved in limonene modification or utilization by the mountain pine beetle-associated pathogen *Grosmannia clavigera*. Appl Environ Microbiol.

[5] Wadke N, Kandasamy D, Vogel H, Lah L, Wingfield BD, Paetz C (2016). The bark-beetle-associated fungus, *Endoconidiophora polonica*, utilizes the phenolic defense compounds of its host as a carbon source. Plant Physiol.

[6] Krokene P. Conifer defense and resistance to bark beetles. In: Vega FE, Hofstetter RW, editors. *Bark beetles: biology and ecology of native and invasive species*. Elsevier; 2015, p. 177–207.

[7] Ranger CM, Biedermann PHW, Phuntumart V, Beligala GU, Ghosh S, Palmquist DE (2018). Symbiont selection via alcohol benefits fungus farming by ambrosia beetles. Proc Natl Acad Sci USA.

[8] Hofstetter RW, Cronin JT, Klepzig KD, Moser JC, Ayres MP (2006). Antagonisms, mutualisms and commensalisms affect outbreak dynamics of the southern pine beetle. Oecologia.

[9] Richard FJ, Poulsen M, Hefetz A, Errard C, Nash DR, Boomsma JJ (2007). The origin of the chemical profiles of fungal symbionts and their significance for nestmate recognition in *Acromyrmex* leaf-cutting ants. Behav Ecol Sociobiol.

[10] Kandasamy D, Gershenzon J, Hammerbacher A (2016). Volatile organic compounds emitted by fungal associates of conifer bark beetles and their potential in bark beetle control. J Chem Ecol.

[11] Biedermann PHW, Kaltenpoth M (2014). New synthesis: the chemistry of partner choice in insect-microbe mutualisms. J Chem Ecol.

[12] Becher PG, Flick G, Rozpedowska E, Schmidt A, Hagman A, Lebreton S (2012). Yeast, not fruit volatiles mediate *Drosophila melanogaster* attraction, oviposition and development. Funct Ecol.

[13] Mansourian S, Stensmyr MC (2015). The chemical ecology of the fly. Curr Opin Neurobiol.

[14] Jeger M, Bragard C, Caffier D, Candresse T, Chatzivassiliou E, Dehnen-Schmutz K, et al. Pest categorisation of *Ips typographus*. EFSA J. 2017;15:4881. 10.2903/j.efsa.2017.5040 PMC7009941 32625323

[15] Wermelinger B (2004). Ecology and management of the spruce bark beetle *Ips typographus*—a review of recent research. Ecol Manag.

[16] Kirisits T. Fungal associates of European bark beetles with special emphasis on the ophiostomatoid fungi. In: Lieutier F, Day KR, Battisti A, Grégoire JC, Evans HF, editors. *Bark and wood boring insects in living trees in europe, a synthesis*. Dordrecht: Springer; 2007, p. 181–236.

[17] Linnakoski R, Wilhelm de Beer ZB, Niemelä P, Wingfield MJ (2012). Associations of conifer-infesting bark beetles and fungi in Fennoscandia. Insects.

[18] Linnakoski R, Mahilainen S, Harrington A, Vanhanen H, Eriksson M, Mehtatalo L (2016). Seasonal succession of fungi associated with *Ips typographus* beetles and their phoretic mites in an outbreak region of Finland. PLoS ONE.

[19] Schmidberger J (1836). Naturgeschichte des apfelborkenkäfers apate dispar. Beitr Zur Obs und Zur Nat der Den Obs schädlichen Insekt.

[20] Harrington TC (2005). Ecology and evolution of mycophagous bark beetles and their fungal partners. Insect-Fungal Assoc. Ecol Evol.

[21] Hanssen HP. Volatile metabolites produced by species of *Ophiostoma* and *Ceratocystis*. In: Wingfield MJ, Seifert KA, Webber JF, editors. *Ceratocystis and ophiostoma: taxonomy, ecology, and pathology*. St. Paul: APS Press; 1993, p. 117–25.

[22] Sprecher E, Kubeczka KH, Ratschko M (1975). Flüchtige terpene in pilzen. Arch Pharm (Weinh).

[23] Cale JA, Collignon RM, Klutsch JG, Kanekar SS, Hussain A, Erbilgin N (2016). Fungal volatiles can act as carbon sources and semiochemicals to mediate interspecific interactions among bark beetle-associated fungal symbionts. PLoS ONE.

[24] Andersson MN, Larsson MC, Schlyter F (2009). Specificity and redundancy in the olfactory system of the bark beetle *Ips typographus*: Single-cell responses to ecologically relevant odors. J Insect Physiol.

[25] Tømmerås BAring (1985). Specialization of the olfactory receptor cells in the bark beetle *Ips typographus* and its predator *Thanasimus formicarius* to bark beetle pheromones and host tree volatiles. J Comp Physiol A.

[26] Lanne BS, Ivarsson P, Johnsson P, Bergström G, Wassgren AB (1989). Biosynthesis of 2-methyl-3-buten-2-ol, a pheromone component of *Ips typographus* (Coleoptera: Scolytidae). Insect Biochem.

[27] Zhao T, Axelsson K, Krokene P, Borg-Karlson AK (2015). Fungal symbionts of the spruce bark beetle synthesize the beetle aggregation pheromone 2-methyl-3-buten-2-ol. J Chem Ecol.

[28] Unelius CR, Schiebe C, Bohman B, Andersson MN, Schlyter F (2014). Non-host volatile blend optimization for forest protection against the European spruce bark beetle, *Ips typographus*. PLoS ONE.

[29] Zhang Schlyter FQ (2003). Redundancy, synergism, and activite inhibitory range of non-host volatiles in reducing pheromone attraction in European spruce bark beetle I*ps typographus*. Oikos.

[30] Andersson MN, Löfstedt C, Newcomb RD (2015). Insect olfaction and the evolution of receptor tuning. Front Ecol Evol.

[31] Six DL (2012). Ecological and evolutionary determinants of bark beetle—fungus symbioses. Insects.

[32] Raffa KF, Grégoire JC, Lindgren BS. Natural history and ecology of bark beetles. In: Raffa KE, editor. *Bark beetles: biology and ecology of native and invasive species*. Academic Press; 2015, p. 1–28.

[33] Six DL, Wingfield MJ (2011). The role of phytopathogenicity in bark beetle–fungus symbioses: a challenge to the classic paradigm. Annu Rev Entomol.

[34] Khadempour L, LeMay V, Jack D, Bohlmann J, Breuil C (2012). The relative abundance of mountain pine beetle fungal associates through the beetle life cycle in pine trees. Microb Ecol.

[35] Moser JC, Perry TJ, Solheim H (1989). Ascospores hyperphoretic on mites associated with *Ips typographus*. Mycol Res.

[36] Solheim H (1991). Oxygen deficiency and spruce resin inhibition of growth of blue stain fungi associated with *Ips typographus*. Mycol Res.

[37] Saerens SMG, Delvaux FR, Verstrepen KJ, Thevelein JM (2010). Production and biological function of volatile esters in *Saccharomyces cerevisiae*. Microb Biotechnol.

[38] Christiaens JF, Franco LM, Cools TL, de Meester L, Michiels J, Wenseleers T (2014). The fungal aroma gene *ATF1* promotes dispersal of yeast cells through insect vectors. Cell Rep.

[39] Luna E, Cranshaw W, Tisserat N (2014). Attraction of walnut twig beetle *Pityophthorus juglandis* (Coleoptera: Curculionidae) to the fungus *Geosmithia morbida*. Plant Heal Prog.

[40] Hulcr J, Mann R, Stelinski LL (2011). The scent of a partner: Ambrosia beetles are attracted to volatiles from their fungal symbionts. J Chem Ecol.

[41] Brand JM, Schultz J, Barras SJ, Edson LJ, Payne TL, Hedden RL (1977). Bark-beetle pheromones—enhancement of *Dendroctonus frontalis* (Coleoptera: Scolytidae) aggregation pheromone by yeast metabolites in laboratory bioassays. J Chem Ecol.

[42] Yang S, Mei XD, Zhang XF, Li YF, She D, Zhang T (2017). Attraction of coffee bean weevil, *Araecerus fasciculatus*, to volatiles from the industrial yeast *Kluyveromyces lactis*. J Chem Ecol.

[43] Bentz BJ, Six DL (2006). Ergosterol content of fungi associated with *Dendroctonus ponderosae* and D*endroctonus rufipennis* (Coleoptera: Curculionidae, Scolytinae). Ann Entomol Soc Am.

[44] Bleiker KP, Six DL (2007). Dietary benefits of fungal associates to an eruptive herbivore: potential implications of multiple associates on host population dynamics. Environ Entomol.

[45] Hammerbacher A, Schmidt A, Wadke N, Wright LP, Schneider B, Bohlmann J (2013). A common fungal associate of the spruce bark beetle metabolizes the stilbene defenses of Norway spruce. Plant Physiol.

[46] Zhao Tao, Kandasamy Dineshkumar, Krokene Paal, Chen Jingyuan, Gershenzon Jonathan, Hammerbacher Almuth (2019). Fungal associates of the tree-killing bark beetle, Ips typographus, vary in virulence, ability to degrade conifer phenolics and influence bark beetle tunneling behavior. Fungal Ecology.

[47] Horntvedt R, Christiansen E, Solheim H, Wang S (1983). Artificial inoculation with *Ips typographus*-associated blue-stain fungi can kill healthy Norway spruce trees. Medd Nor Inst Skogforsk.

[48] Kallenbach M, Oh Y, Eilers EJ, Veit D, Baldwin IT, Schuman MC (2014). A robust, simple, high-throughput technique for time-resolved plant volatile analysis in field experiments. Plant J.

[49] Anderbrant O, Schlyter F, Birgersson G (1985). Intraspecific competition affecting parents and offspring in the bark beetle *Ips typographus*. Oikos.

[50] Andersson MN, Larsson MC, Svensson GP, Birgersson G, Rundlöf M, Lundin O (2012). Characterization of olfactory sensory neurons in the white clover seed weevil, *Apion fulvipes* (Coleoptera: Apionidae). J Insect Physiol.

[51] Andersson MN, Schlyter F, Hill SR, Dekker T (2012). What reaches the antenna? How to calibrate odor flux and ligand-receptor affinities. Chem Senses.

[52] Hallberg E (1982). Sensory organs in *Ips typographus* (Insecta: Coleoptera): fine structure of antennal sensilla. Protoplasma.

