# Supplementary material for: Volatile organic compounds influence the interaction of the Eurasian spruce bark beetle (Ips typographus) with its fungal symbionts
Source: ISME J. 2019 Mar 14;13(7):1788–800. doi: 10.1038/s41396-019-0390-3 (PMC6775991; doi:10.1038/s41396-019-0390-3)
Supplement: Supplementary file 1 — Supplemental figure 1 [file 41396_2019_390_MOESM1_ESM.docx]

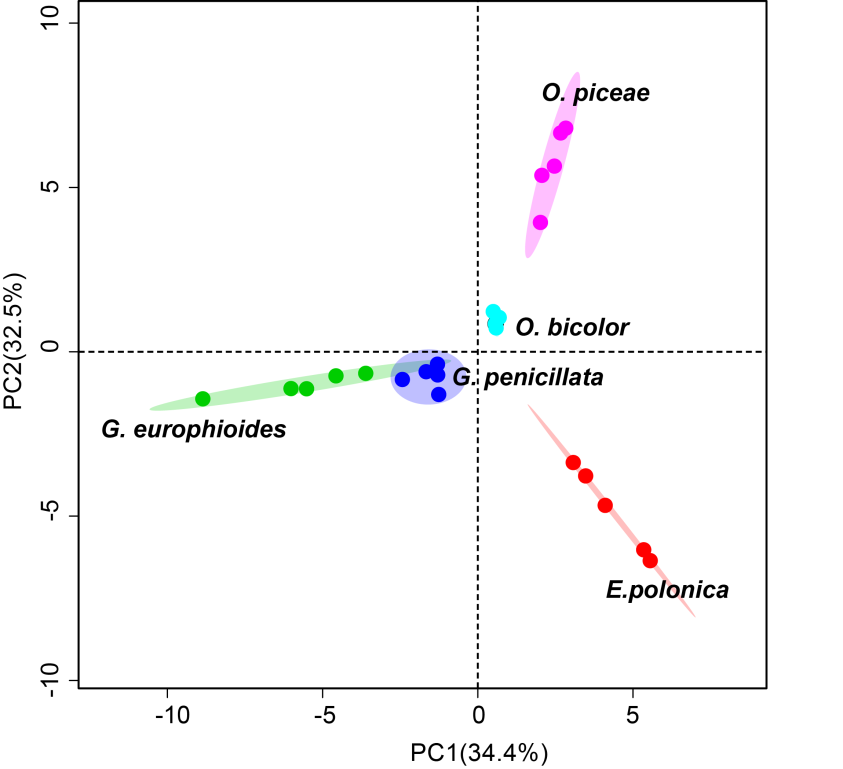


***Figure S1*: Volatile profiles of fungi associated with *Ips typographus* show that their blends can be readily distinguished.** Principal component analysis (PCA) based on the composition of volatiles emitted by each fungus growing on potato dextrose agar. PC1 and PC2 represent 34.4% and 32.5% of total variation, respectively. Ellipses denote a 95 percent confidence interval around each species. The PCA plot was generated by using MetaboAnalyst 3.0 software with normalized data.
